# Unraveling Cancer’s Wnt Signaling: Dynamic Control through Protein Kinase Regulation

**DOI:** 10.3390/cancers16152686

**Published:** 2024-07-28

**Authors:** Deniz Tümen, Philipp Heumann, Julia Huber, Nele Hahn, Celina Macek, Martha Ernst, Arne Kandulski, Claudia Kunst, Karsten Gülow

**Affiliations:** Department of Internal Medicine I Gastroenterology, Hepatology, Endocrinology, Rheumatology, Immunology, and Infectious Diseases, University Hospital Regensburg, 93053 Regensburg, Germany; deniz.tuemen@ukr.de (D.T.); nele.hahn@stud.uni-regensburg.de (N.H.);

**Keywords:** Wnt signaling, canonical, non-canonical, β-catenin, planar-cell-polarity, Ca^2+^ signaling, kinases, phosphorylation, cancer, cancer-therapy

## Abstract

**Simple Summary:**

The Wnt signaling pathway plays a pivotal role in governing developmental processes and maintaining stem cell characteristics, while also exhibiting significant implications in cancer pathogenesis. While initially characterized in colorectal cancer, aberrations in Wnt signaling are pervasive across various cancer types. Both intrinsic and extrinsic factors modulate Wnt signaling, mainly influencing the strictly coordinated kinase cascade within Wnt signaling. Understanding the complex interplay of canonical and non-canonical Wnt pathways and their potential dysregulation in disorders holds promise for the development of innovative therapeutic strategies in cancer treatment.

**Abstract:**

Since the initial identification of oncogenic Wnt in mice and Drosophila, the Wnt signaling pathway has been subjected to thorough and extensive investigation. Persistent activation of Wnt signaling exerts diverse cancer characteristics, encompassing tumor initiation, tumor growth, cell senescence, cell death, differentiation, and metastasis. Here we review the principal signaling mechanisms and the regulatory influence of pathway-intrinsic and extrinsic kinases on cancer progression. Additionally, we underscore the divergences and intricate interplays of the canonical and non-canonical Wnt signaling pathways and their critical influence in cancer pathophysiology, exhibiting both growth-promoting and growth-suppressing roles across diverse cancer types.

## 1. Introduction

In the early 1980s, Wnt signaling was independently discovered in two distinct organisms. During the genetic screening of *Drosophila* for body pattern defects, the defective *WN1* gene was identified as the species were forming “wg” (short for wingless) phenotypes [1]. Later, Roel Nusse and Harold Varmus conducted an experimental study wherein they exposed mice to the mouse mammary tumor virus (MMTV) to induce genetic mutations and to elucidate the specific mutated genes responsible for the development of breast tumors. In the course of their investigation, they successfully isolated and characterized a previously unidentified murine proto-oncogene, which they named “int1” (short for integration 1) [2]. The term “Wnt” became established as a result of the combination of the Drosophila gene “Wingless” (Wg) polarity and the vertebrate homolog “Integrated 1” (Int-1) [3,4,5]. The Wnt signaling pathway exhibits a remarkable degree of evolutionary conservation and exerts substantial influence on embryogenesis, tissue homeostasis, and regenerative processes in adult tissue [6,7]. Furthermore, it plays a pivotal role in maintaining genetic stability, while also governing critical cellular processes such as fate determination, differentiation, apoptosis, cell migration, and the maintenance of stem cells [8]. Dysregulation of Wnt signaling is closely associated with a spectrum of disorders, including embryogenic malformations, degenerative diseases, diabetes, autoimmune disorders, and cancer [9,10,11].

The Wnt signaling cascade is differentiated into four distinct categories: Firstly, the canonical pathway is characterized by the involvement of the transcriptional co-activator cadherin-associated protein β (β-catenin) and the TCF/LEF (T cell factor/lymphoid enhancer factor) family of transcription factors. It is therefore also stated as the β-catenin-dependent canonical Wnt signaling pathway. Secondly, the more recently discovered Wnt/STOP signaling pathway counts to the canonical Wnt signaling and is independent of β-catenin. It primarily influences the stabilization and degradation processes of specific proteins within the cell. Wnt/STOP signaling, therefore, has a critical role in protecting proteins, such as c-MYC, from GSK3-dependent polyubiquitination and degradation. It is proposed that decelerated protein degradation is beneficial for cell growth in preparation for cells to divide [12]. Further, stabilization of proteins via Wnt/STOP triggers an LRP6-DVL-dependent signaling cascade which is required for proper mitosis regulation and chromosome segregation. Intact Wnt/STOP signaling prevents chromosome missegregation and aneuploidy [13].

The third and fourth categories encompass the non-canonical Wnt signaling pathways, which do not involve β-catenin as well. Those are in particular, the planar cell polarity (PCP) pathway and the Wnt calcium pathway (Wnt-Ca^2+^) [14].

The Wnt signaling pathways are responsible for the regulation of fundamental cellular processes, which necessitate tight regulation through various mechanisms. Within the signaling cascade, specific phosphorylation events intricately regulate aspects such as protein activity, binding affinities, and protein stability [15]. This review aims to consolidate an overview of the complex nature of Wnt signaling, elucidate the molecular implications, and provide insights into the regulation of the various kinases associated with the regulation or dysregulation of the Wnt signaling pathway. Especially mentioning the latter, the Wnt signaling pathway is also known for its dual role in cancer, acting as a driver of tumor growth in some cancers while suppressing growth in others. This duality represents a critical but often underappreciated aspect of Wnt signaling. Understanding these opposing roles is essential for developing targeted cancer therapies, an aspect we would also like to examine in more detail.

## 2. Canonical and Non-Canonical Wnt Signaling: A Comprehensive Categorization

### 2.1. Canonical Wnt/β-Catenin Pathway

**Figure 1:**
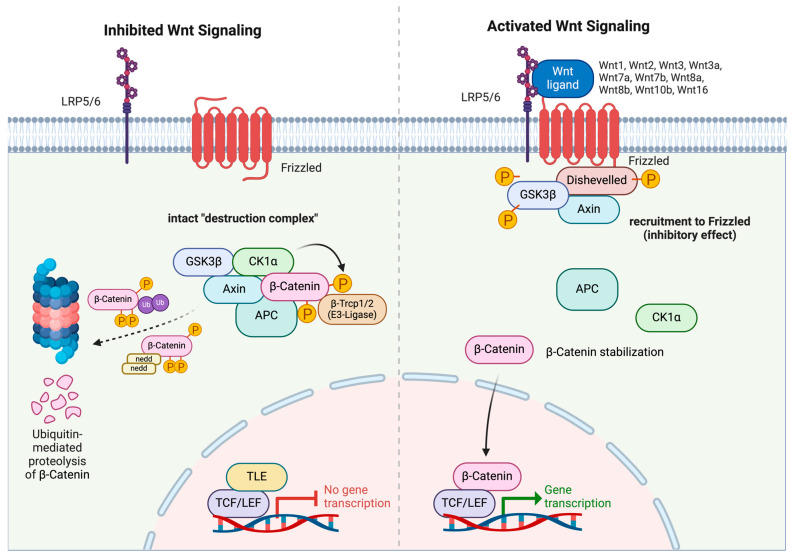
Molecular mechanisms of inhibited and activated canonical Wnt/β-catenin signaling pathway. In the absence of Wnt ligands, LRP5/6 and Frizzled receptors remain spatially separated. The “destruction complex” consisting of Axin, APC, GSK3β, and CK1α phosphorylates β-catenin and marks it for β-TrCP1/2-dependent ubiquitination and neddylation for subsequent proteasomal degradation. Upon Wnt ligand binding to Frizzled, LRP5/6 and Frizzled receptors interact and subsequently recruit Dishevelled, which in turn recruits GSK3β and Axin and favors their phosphorylation. In the absence of active GSK3β and Axin, the “destruction complex” cannot form. β-catenin is stabilized and diffuses into the nucleus, where it interacts with TCF/LEF and induces transcriptional activation of downstream genes. This illustration was created with BioRender.com (accessed on 24 July 2024).

The canonical Wnt pathway is typically characterized by a high degree of evolutionary conservation. Activation is primarily achieved through the binding of extracellular Wnt ligands (Wnt1, Wnt2, Wnt2b, Wnt3, Wnt3a, Wnt6, Wnt7a, Wnt7b, Wnt8a, Wnt8b, Wnt10b, or Wnt16) to the corresponding FZD (Frizzled) receptor and the low-density co-receptors LRP5 or LRP6 (LRP5/6) [6,16,17]. Upon activation, the canonical Wnt pathway leads to the stabilization of β-catenin and promotes its translocation to the nucleus (Figure 1; right) [18,19]. The translocation of β-catenin into the nucleus still represents a controversial process [19]. The phenylalanine–glycine (FG) repeats of nucleoporins typically inhibit the nuclear entry of proteins exceeding 40 kDa in size [20]. According to the conventional nuclear transport model, proteins containing a nuclear localization sequence (NLS) bind to Importin-α, which then associates with the nuclear pore complex (NPC) and traverses the nuclear pore through its interaction with Importin-β. Upon entry into the nucleus, the protein cargo is released by the activity of Ran GTPases [21].

It was previously thought that β-catenin lacks a discernible NLS. However, recent research suggests the presence of an NLS-like domain within the C-terminus of β-catenin, which is crucial for its nuclear translocation. Deleting the C-terminal domain, as opposed to the N-terminal domain or the central armadillo repeats, significantly impairs nuclear transport [22]. Additionally, it has been demonstrated that the nuclear import of β-catenin is contingent upon the functional activity of Ran GTPases, further supporting the hypothesis that this nuclear transport process is mediated by Importin-α and the NPC [22]. There, β-catenin subsequently stimulates target genes, including *CYCLIN D1*, *c-MYC*, *PDK*, *MTC-1*, *MMP7*, fibronectin, *COX-2,* and *AXIN-2* [23,24,25,26] promoting processes such as cell proliferation, survival, differentiation, and cell migration [11] and influencing inflammatory, glycolytic, and circadian rhythmic pathways [27]. To date, exclusively the increased stabilization, high quantity, and enhanced translocation of β-catenin into the nucleus have been associated with carcinogenesis. Consequently, numerous studies are being conducted to counteract tumor development by specifically targeting β-catenin’s stability, nuclear translocation, and interaction with nuclear proteins [28]. Conversely, there are no reports available stating that insufficient levels of β-catenin contribute to tumorigenesis.

In the absence of Wnt ligands, the transmembrane receptors FZD and LRP5/6 are separately localized at the plasma membrane. This spatial separation allows for the formation of a so-called destruction complex, which is composed of adenomatous polyposis coli (APC), Axin, casein kinase 1 (CK1), and glycogen synthase kinase 3β (GSK3β) [29]. This complex, primarily mediated by Axin and Dishevelled (Dvl), actively captures and phosphorylates β-catenin. Phosphorylation effectively promotes the ubiquitination and neddylation, and commences the degradation of β-catenin [30]. As a consequence, Transducin-like enhancer (TLE), an antagonist that binds to the transcription factors T-cell factor/lymphoid enhancer-binding factor (TCF/LEF), exerts an inhibitory influence on the transcription of the respective target genes (Figure 1; left) [31].

However, binding of the Wnt ligands to their transmembrane receptors mediates the colocalization and complexation of FZD and LRP5/6. The destruction complex then undergoes relocalization to the plasma membrane, since Dvl, an FZD-interacting protein, facilitates the docking of Axin and GSK3β to FZD receptors to subsequently engage the phosphorylation of LRP5/6 [32]. This process inhibits the destruction complex from degrading β-catenin. The accumulation of cytosolic β-catenin favors its translocation to the nucleus, where it interacts with TCF/LEF [33], thereby promoting the transcription of target genes [34]. This dynamic cytoplasmic–nuclear shuttling of β-catenin represents a pivotal hallmark in the activation of the Wnt/β-catenin signaling pathway.

### 2.2. Non-Canonical Wnt/PCP and Wnt/Ca^2+^ Signaling Pathways

**Figure 2:**
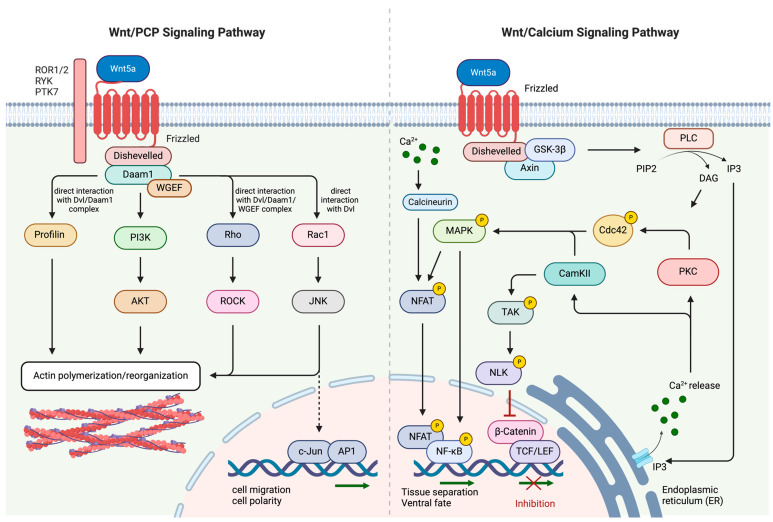
Signaling cascade of non-canonical Wnt/PCP and Wnt/Ca^2+^ pathway. The Wnt/PCP is activated upon specific Wnt ligand binding to several transmembrane receptors including ROR1/2, RYK, PTK7, and Frizzled. This results in the activation of several GTPases through direct interaction with the Dvl/Daam1/WGEF complex (Roh), with the Dvl/Daam1 complex (Profilin, PI3K), or with Dvl (Rac1) alone. Activated GTPases either induce formation, polymerization, or reorganization of the cytoskeleton. Wnt/Ca^2+^ signaling is activated upon binding of specific Wnt ligands to the corresponding Frizzled receptor. IP3-mediated release of Ca^2+^ ions from the endoplasmic reticulum into the cytosol positively modulates the kinase activity of PKC which in turn phosphorylates and activates Cdc42. Cdc42 and CamKII together phosphorylate and activate p38 MAPK. Activated p38 MAPK and Ca^2+^-dependent activation of calcineurin are essential for the phosphorylation and activation of NFAT [44]. NF-κB is also a direct target of MAP kinases that phosphorylate and activate it. Transcriptional activation of both, NFAT and NF-κB regulate tissue separation and ventral fate during embryogenesis. On the other hand, Ca^2+^ release is also responsible for CamKII activation. In a subsequent phosphorylation cascade, activated TAK and NLK prevent the assembly of β-catenin and TCF/LEF, thus, inhibiting the β-mediated transcriptional activation of downstream genes. Black arrows indicate the activation of the respective downstream proteins within the Wnt-signaling cascade. Green arrows indicate transcriptional activation of genes. Red “inhibition” arrow indicated the inhibition of proteins. This illustration was created with BioRender.com (accessed on 24 July 2024).

Activation of the non-canonical Wnt pathway occurs upon binding of specific Wnt ligands, including Wnt3a, Wnt4, Wnt5a, Wnt5b, Wnt6, Wnt7b, and Wnt11 to their corresponding receptors [35]. Non-canonical Wnt signaling can be subdivided into two distinctive pathways: the planar cell polarity (PCP)/Wnt pathway and the Wnt/Ca^2+^ pathway (Figure 2) [29]. The PCP pathway plays a critical role in regulating cellular processes such as polarization, adhesion, stem cell maintenance, embryonic development, and cellular migration [36]. The Wnt ligands interact with receptors from the FZD receptor family, receptor tyrosine kinase-like orphan receptor 1 or 2 (ROR1 or ROR2), receptor-like tyrosine kinase (RYK), and protein tyrosine kinase 7 (PTK7) [37,38]. Wnt ligand-mediated colocalization of FZD and ROR/RYK receptors allows for the binding of Dvl to FZD and the subsequent association and activation of the Dvl-associated activator of morphogenesis 1 (Daam1). This complex, in turn, activates small GTPase proteins such as Ras-related C3 botulinum toxin substrate 1 (Rac1) and Ras-homologous (Rho) [39]. In the subsequent step, Rho and Rac1 activate Rho-associated protein kinase (ROCK) and c-Jun N-terminal kinase (JNK) [36,40]. Another protein directly regulated by Dvl and Daam1 is Profilin, a ubiquitous protein that is essential for the development of the cellular cytoskeleton [41,42]. Profilin, ROCK, and JNK collectively regulate cell polarity and cell migration via the formation of the cytoskeleton [43]. Aberrant regulation of this signaling pathway and the resulting increase in cellular migration have also been implicated in cancer.

The Wnt/Ca^2+^ signaling pathway, on the other hand, is mainly involved in ventral cell fate and tissue separation during embryogenesis. The binding of a Wnt ligand to its cognate FZD receptor triggers a transient increase in intracellular concentrations of specific signaling molecules, specifically inositol 1,4,5-triphosphate (IP3), 1,2-diacylglycerol (DAG), and Ca^2+^ [45]. IP3 and DAG are converted from a plasma membrane-resident phospholipid, namely phosphatidyl inositol 4,5-bisphosphate (PIP2) through enzymatically active phospholipase C (PLC) [46]. PLC is also localized on the plasma membrane and becomes activated upon FZD receptor–Wnt ligand interaction. IP3, once produced, diffuses through the cytosol and associates with calcium channels situated on the membrane of the endoplasmic reticulum (ER), leading to the release of calcium ions [47]. Ca^2+^ ions, along with the ubiquitously expressed eukaryotic protein calmodulin, activate calcium calmodulin-dependent protein kinase II (CaMKII) [48], whereas DAG and cytosolic Ca^2+^ positively influence protein kinase C (PKC) activity [49]. CaMKII and PKC are both capable of stimulating further GTPases, including Cdc42 which, in turn, interacts with mitogen-activated protein kinases (MAP3K10, MAP3K11) to recruit various transcription factors (e.g., NF-κB and CREB) for the stimulation of downstream genes involved in embryonic tissue separation and gastrulation [50]. Moreover, CaMKII acts as a modulator of MAP3K7, also known as TAK1. TAK1-mediated activation of the nemo-like kinase (NLK) results in the displacement of DNA-associated β-catenin/TCF/LEF [51]. This signaling cascade is to be understood as a direct inhibition of the canonical Wnt signaling pathway [52]. Similarly, IP3-induced Ca^2+^ release can activate phosphatase calcineurin (CN), which triggers cytoplasmic protein nuclear factor associated with T cells (NFAT) through dephosphorylation. Interestingly, activated NFAT was shown to directly interact with Dvl to influence the interaction between β-catenin and Dvl in a competitive manner [44]. Activated NFAT thereby negatively modulates the canonical Wnt signaling pathway and the associated proliferation of cells while being responsible for the expression of several genes involved in ventral cell differentiation during embryogenesis [53].

### 2.3. Protein Structure of Wnt Ligand and FZD Receptor Binding

**Figure 3:**
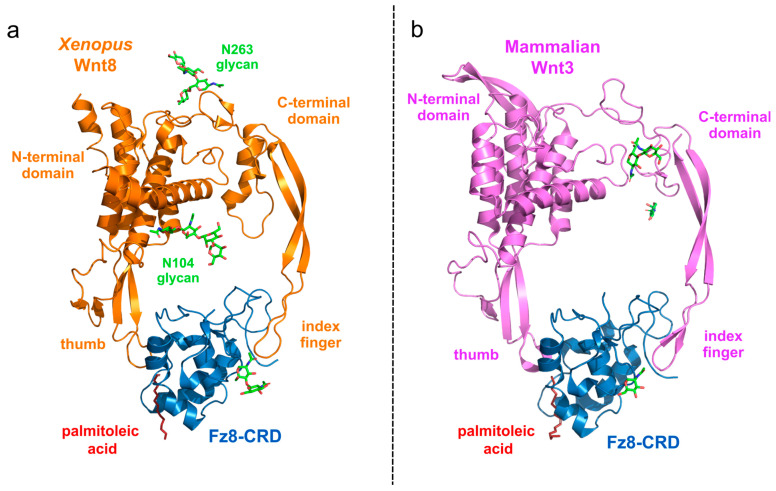
Protein structure of Wnt ligand and FZD receptor complexation. (**a**) Crystal structure of complexed *Xenopus* xWnt8 from and murin Fz8-cysteine-rich domain (CRD) was resolved by Janda et al. [54] revealing a novel protein fold and ligand–receptor interaction. The hand-like protein structure of xWnt8 resembles a “thumb” and an “index finger” enclosing the Fz8-CRD domain and enabling a close ligand-receptor cross-reactivity. Posttranslational modification on xWnt8 with palmitoleic acid shows the importance of its existence for proper ligand–receptor interaction. Crystal structure was derived from the Protein database (PDB, code: 4F0A) and illustrated with PyMol. (**b**) First crystal structure of complex mammalian Wnt3 ligand and murine Fz8-CRD receptor was resolved by Hirai et al. [55]. Direct comparison between xWnt8 and mammalian Wnt8 reveals the structural similarity which suggests that most of the structurally uncharacterized Wnt ligands bind to their respective receptors with the same principle. Crystal structure was derived from the Protein database (PDB, code: 6AHY) and illustrated with PyMol (version 2.6).

The structural characterization of Wnt protein family members remains relatively limited, as the partly O-lipidated serine residues render Wnt proteins highly hydrophobic and difficult to purify. The first resolved structure of a Wnt protein was that of *Xenopus* Wnt8 (xWnt8) in complex with the mouse FZD8 (original nomenclature, Fz8) cysteine-rich domain (CRD). The first crystal structure of xWnt8/Fz8 complexation revealed a novel protein fold and highlighted the essential role of lipidation in direct FZD binding (Figure 3a) [54]. This structural analysis demonstrated that Wnts interact with FZD at two distinct sites located on opposite faces of the CRD. XWnt8 resembles a hand-like protein structure with “thumb” and “index” fingers extending to interact with the Fz8-CRD at two separate binding sites. At the first binding site, a palmitoleic acid lipid group, emanating from Serine 187 at the tip of the Wnt “thumb”, inserts into a deep groove in the Fz8-CRD. The second binding site involves the conserved tip of the Wnt “index finger”, which forms hydrophobic interactions on the opposite side of the Fz8-CRD. The conserved amino acids at both interfaces likely promote ligand–receptor cross-reactivity [54]. Although the Wnt protein family comprises 19 members in mammals, the structure of only one mammalian Wnt protein has been so long, namely mammalian Wnt3 complexed with murine Fzd8 (Figure 3b) [55]. The structural similarity between xWnt8 and mammalian Wnt3 suggests that most of the structurally yet uncharacterized Wnt ligands bind to their respective receptors with the same principle.

The canonical Wnt signaling pathway is mainly stimulated through Wnt-mediated activation and spatial complexation of LRP5/6 and FZD receptors. Wnt ligands primarily influencing receptors of the canonical Wnt signaling pathway include Wnt1, Wnt2, Wnt2b, Wnt3, Wnt3a, Wnt4, Wnt5a, Wnt6, Wnt7a, Wnt9a, Wnt10a, and Wnt10b [56]. The non-canonical Wnt/PCP and Wnt/Ca^2+^ signaling pathways are mainly stimulated through Wnt-mediated activation and spatial complexation of ROR1/2 and FZD receptors. Wnt ligands primarily activating receptors of the Wnt/PCP and Wnt/Ca^2+^ signaling pathway include Wnt1, Wnt2, Wnt5a, Wnt5b, and Wnt11 (Table 1) [56].

## 3. Wnt Signaling in Cancer

### 3.1. Mutational Influence on Canonical Wnt/β-Catenin Signaling

**Figure 4:**
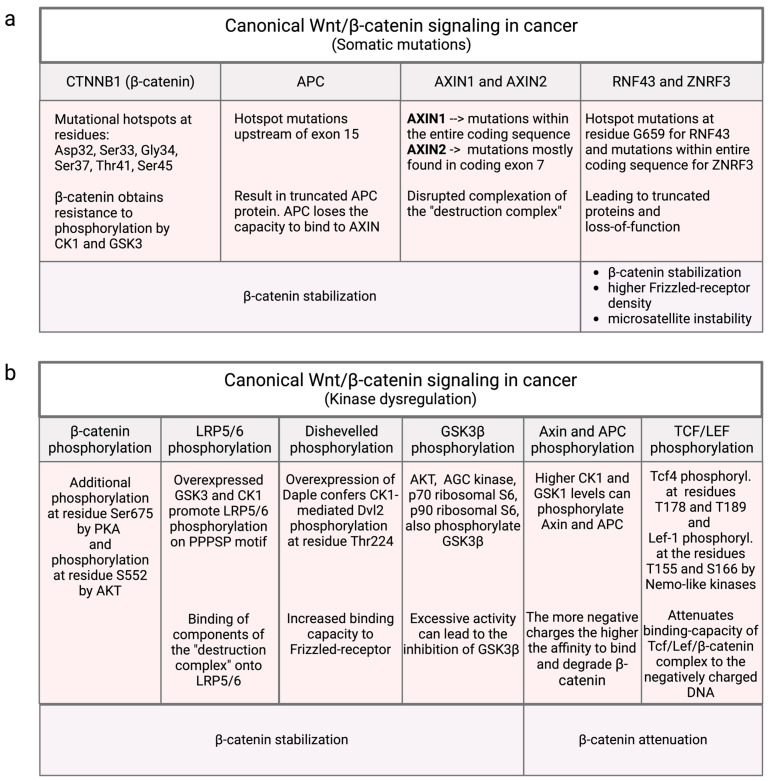
Canonical Wnt/β-catenin signaling in cancer and distinction between mutational and non-mutational causes. (**a**) Mutations within the respective genes of β-catenin (CTNNB1), APC, Axin1/2, RNF43, and ZNRF3 have all in common to stabilize β-catenin and ubiquitously activate transcription of downstream genes. Mutations in RNF and ZNRF3 mutations additionally lead to higher FZD-receptor density and DNA-microsatellite instability. (**b**) Kinases with enhanced activities uncontrollably phosphorylate components of the canonical Wnt signaling pathway, including β-catenin, LRP5/6, Dishevelled (Dvl), GSK3β, Axin1/2, and TCF/LEF. Excessive phosphorylation of β-catenin, LRP5/6, Dvl, and GSK3β stabilize β-catenin while phosphorylation of Axin, APC, and TCF/LEF attenuates β-catenin. However, decreased activities and lower phosphorylation rates for Axin1/2 and TCF/LEF can also result in the stabilization of β-catenin. This illustration was created with BioRender.com (accessed on 24 July 2024).

CTNNB1 gene mutations, responsible for encoding β-catenin, are intricately linked with a spectrum of malignancies, encompassing hepatocellular carcinoma (HCC) [57], pancreatic cancer, colorectal cancer (CRC), gastroesophageal junction carcinoma, and gastric adenocarcinoma (Table 2). Notably, a mutational hotspot is concentrated in exon 3 of CTNNB1, specifically around the phosphorylation sites targeted by the destruction complex [58]. Mutations in these phosphorylation sites, namely Ser33, Ser37, Thr41, and Ser45, confer resistance to phosphorylation by CK1α or GSK3β, resulting in the stabilization of β-catenin (Figure 4a). In addition, frequent mutations in Asp32 and Gly34, which are critical for proper binding to β-TrCP E3 ubiquitin–ligase complex, disrupt the ubiquitination of β-catenin [58]. This mutational event has been identified in a variety of solid tumors and is considered a potential driver mutation, accounting for 3.3–10.4% of all documented β-catenin mutations [59,60].

The APC protein serves as a substantial scaffold protein encompassing multiple domains for its interaction with binding partners within the destruction complex, resulting in the facilitated degradation of β-catenin [61]. APC is the second most commonly mutated gene in CRC after *TP53*, with mutations occurring in more than 50% of patients [62]. In colon cancer, a significant proportion of mutations are predominantly located upstream of exon 15, resulting in the translation of truncated APC proteins [63]. These truncations typically retain substantial segments of the β-catenin domain while losing the capacity to bind to AXIN, thereby slightly increasing Wnt signaling levels. Consequently, the prevalent APC mutation identified in colorectal cancer (CRC) retains some degree of β-catenin binding capacity, thus preventing the induction of maximal Wnt signaling [64]. APC mutations are also identified in approximately 13–15% of cases in uterine endometrial cancer, stomach cancer, and skin cutaneous melanoma [61].

Both AXIN1 and AXIN2 function as scaffold proteins within the destruction complex to modulate the levels of β-catenin. In HCC, AXIN1 mutations are detected in approximately 8% of patients, and approximately 14% of uterine endometrial cancers, and AXIN2 is mutated in about 5% of CRC patients [61,65]. Although these two proteins exhibit a considerable degree of homology, their mutation patterns diverge significantly. For instance, AXIN1 mutations encompass the entire coding sequence and exhibit heterogenic mutations across various tumor types. On the other hand, AXIN2 consistently displays a frameshift mutation in exon 7 [66].

The ubiquitin ligases RNF43 and ZNRF3 collectively contribute to a negative feedback loop, orchestrating the internalization and degradation of FZD receptors, normally reducing Wnt signaling. Notably, in CRC and endometrial cancer, a prominent hotspot mutation at position G659 triggers a frameshift mutation, ultimately yielding a truncated variant of RNF43 [67]. Research underscored the significance of N-terminal truncating mutations within RNF43 for driving increased β-catenin signaling. However, these mutations also increase the vulnerability towards PI3K/mTOR inhibition [68] and BRAF/EGFR therapy [69]. Interestingly, the loss-of-function mutations in RNF43 have also been linked to microsatellite instability (MSI), a distinguishing hallmark particularly prevalent in CRC tumors [70]. Mutations in ZNRF3 have been found in uterine and skin cancer. However, more extensive studies are required to investigate its inactivating mutations [61].

### 3.2. Non-Mutational Influences in Canonical Wnt/β-Catenin Signaling

#### 3.2.1. β-Catenin Phosphorylation

As previously elucidated, the stability of β-catenin is governed by the Wnt-dependent assembly of the destruction complex. Upon examining the intricate protein interactions between β-catenin and the constituents of the destruction complex, it becomes evident that the precise regulation of degradation or stabilization of β-catenin is governed by several phosphorylation events.

The N-terminus of cytosolic β-catenin undergoes constitutive phosphorylation through a dual-kinase mechanism orchestrated by Axin, which is the scaffold protein of the destruction complex. Axin contains binding sites for β-catenin, CK1, GSK3β, and other factors crucial for Wnt-dependent signaling [71]. Members of the CK1 family initiate the phosphorylation of β-catenin at serine 45 (pS45). This initial phosphorylation is a prerequisite for subsequent phosphorylation by GSK3 at residues Ser33, Ser37, and Ser41 [30,72]. It is believed that β-catenin phosphorylation at residues Ser33 and Ser37 is recognized by the β-TrCP1 E3 ubiquitin–ligase complex, which leads to ubiquitination of β-catenin and prompt degradation by the 26S proteasome [73]. New studies revealed β-catenin degradation to be ubiquitin-independent, either by blocking or depleting the β-TrCP1 E3 ubiquitin-ligase complex in HEK293T cells [74]. Furthermore, β-catenin can be neddylated through its interaction with NEDD8 and β-TrCP2. Consequently, neddylated β-catenin undergoes proteasomal degradation. However, the authors also stated TrCP-independent neddylation and proteasomal degradation of β-catenin [74].

Mutations or aberrant regulation affecting Axin or the N-terminal phosphorylation sites of β-catenin have been observed in various human cancers. These mutations have been associated with increased β-catenin stability [75,76,77].

While N-terminal β-catenin phosphorylation by CK1 and GSK3β is well-established, recent investigations have unveiled additional kinases playing a contributory role in regulating β-catenin signaling. Notably, phosphorylation of Ser675 by protein kinase A (PKA) has been implicated in enhancing β-catenin transcriptional activity by increasing its stability (Figure 4b) [78]. Likewise, phosphorylation of β-catenin at S552 by protein kinase B (AKT) stabilizes β-catenin and enhances nuclear transport and transcriptional activation [79].

#### 3.2.2. LRP5/6 Phosphorylation

Wnt ligands initiate the formation of the receptor complex comprising the FZD receptor and the LRP5/6 co-receptor. Upon Wnt ligand binding and receptor complexation, LRP6 is phosphorylated and subsequently recruits Axin to its intracellular domain [80,81]. The removal of Axin from the destruction complex results in the unavailability of Axin as a scaffold protein. Consequently, the formation of the destruction complex is impeded, which results in the stabilization and cytosolic accumulation of β-catenin. Five identical proline-rich PPPSP motifs were identified within the intracellular domain of LRP6, which are functionally indispensable for Wnt signal transduction [82,83]. These PPPSP residues (which are conserved across all species), serve as docking sites for Axin binding. The mutation of the respective serine residues exerts the destabilization of β-catenin [15].

Broadly, Wnt ligand binding to the FZD receptor triggers the phosphorylation of LRP6 creating a binding site for Axin. It has been demonstrated that incubation of cells with a Wnt-conditioned medium induced rapid and pronounced phosphorylation of the intracellular PPPSP motif on LRP6.

GSK3β has been identified as a participant in phosphorylating PPPSP [84]. In vivo, overexpression of GSK3β promoted LRP6 phosphorylation, whereas GSK3β inhibition prevented it. CK1 has also been implicated in LRP5/6 phosphorylation on the same peptide motif. Expression of dominant-negative CK1 selectively inhibits LRP6 phosphorylation, and CK1 phosphorylation is crucial for the recruitment of Axin by LRP6 [85].

#### 3.2.3. Dvl Phosphorylation

The Dishevelled protein family, encompassing Dvl1–3, serves as a conserved positive regulator of canonical Wnt signaling. The translocation of Dvl to the membrane, coupled with the subsequent recruitment of binding partners, results in the disruption of the destruction complex and the stabilization of β-catenin. Upon Wnt ligand binding, Dvl interacts with the FZD receptor and becomes phosphorylated. Multiple kinases are postulated to be involved in the phosphorylation of Dvl, including CK1, Casein kinase 2, and PAR1 [86].

Studies have also shown that Dvl-associating protein with a high frequency of leucine residues (Daple) is a essential component for Wnt signaling. Upon interaction, Daple confers CK1 the capability of phosphorylating Dvl. Daple overexpression induced CK1-mediated Dvl2 phosphorylation at Thr224. Daple mutations lacking the carboxyl-terminal motif to associate with Dvl, retain the ability to interact with CK1, while CK1 loses the capability to phosphorylate Dvl [87].

#### 3.2.4. GSK3β Phosphorylation

The regulatory mechanism of GSK3β is notable for its distinct characteristics. Unlike the majority of protein kinases involved in signal transduction, GSK3 maintains constitutive active and only undergoes inactivation in response to various signaling events. GSK3β represents a substrate in a broad range of signaling pathways, such as the PI3K/Akt, Hedgehog, cyclic adenosine monophosphate (cAMP), MAPK, transforming growth factor-beta (TGF-β), and Wnt signaling pathways [88,89,90]. The respective cellular processes influenced by these signaling pathways are cell proliferation, differentiation, apoptosis, cell cycle, immune response, and organ development [90,91,92,93,94]. Given its extensive functional repertoire, mutations or dysregulation of GSK3β are implicated in numerous diseases [27,95,96]. Pertaining to Wnt signaling, various studies have shown that Wnt-activated LRP6 can directly inhibit GSK3 function. These studies propose that Wnt ligands induce the phosphorylation of the PPPSP motif within LRP6. Phosphorylated LRP6, in turn, depicts a pseudo-substrate of GSK3 that directly competes with GSK binding to the N-terminus of β-catenin [97,98].

The two isoforms GSK3α and GSK3β are often considered as one protein due to their high sequence homology. Through direct phosphorylation of serine 21 in GSK3α and serine 9 in GSK3β, GSK3 activity in general can also be inhibited [99,100]. Kinases that are involved in GSK3 phosphorylation include AKT, AGC kinase, p70 ribosomal S6 kinase, p90 ribosomal S6 kinase, and p38 mitogen-activated protein kinase (MAPK) [100,101]. Excessive activity of these kinases can lead to the inhibition of GSK3 and, consequently, to activation of β-catenin.

In addition to kinases, phosphatases can also have a significant influence on the regulation of the Wnt signaling pathway. Phosphatases dephosphorylate proteins and thus represent the counterpart to kinases. Of note, the balance of phosphorylation and dephosphorylation between kinases and phosphatases is a complex and tightly regulated process. The protein phosphatase 2A (PP2A) family plays a crucial role in regulating multiple signaling pathways involved in tumorigenesis, stem cell maintenance, and self-renewal [102,103]. Numerous studies identify PP2A as a negative regulator of the Wnt signaling pathway. Studies suggest that PP2A does not directly influence β-catenin. Instead, one of its target proteins is GSK3β. PP2A dephosphorylates GSK3β at Ser9, resulting in GSK3β activation [104]. The activated GSK3β subsequently phosphorylates β-catenin, leading to its degradation [102]. Therefore, PP2A most likely exerts inhibitory effects on Wnt signaling, which can be lost if PP2A is downregulated or dysfunctional.

In summary, it is evident that GSK3β is responsible for both the activation of the Wnt signaling pathway (via phosphorylation of the PPPSP motif on LRP5/6) and its inhibition (through direct and indirect destabilization of β-catenin). Therefore, GSK3β has a dual function in regulating β-catenin, contributing to both its stabilization and degradation. Lithium, a widely recognized GSK3β inhibitor, is commonly used in the treatment of bipolar disorder and cancerogenous disorders [105,106]. Despite GSK3β inhibition, clinical observations do not reveal significant therapeutical effects as might be expected [107,108]. This apparent contradiction can be attributed to the complex regulatory mechanisms that apparently control β-catenin stability. The equilibrium between β-catenin stabilization and degradation, mediated by GSK3β, is finely balanced, allowing lithium’s inhibitory effects to occur without causing major functional abnormalities. Furthermore, the wide range of GSK3β substrates within the Wnt signaling network and related cellular processes may compensate and offset the impact of, and through this, the inhibitory effect on GSK3β.

#### 3.2.5. Axin and APC Phosphorylation by CK1 and GSK3

The recruitment of β-catenin to the Axin/APC destruction complex is governed and regulated by a sequence of orchestrated phosphorylation events [109]. β-catenin is a member of the Armadillo (ARM) repeat protein superfamily. The central region of each ARM repeat consists of approximately 42 residues, organized into three helices that are additionally arranged in a triangular shape. Collectively, the ARM repeats to create a superhelix characterized by a lengthy, positively charged groove [109]. Of note, these positive charges determine the binding efficacy of β-catenin to many of its negatively charged interaction partners, including cadherin adhesion receptor, Axin, APC, and TCF DNA binding factors [33,110]. The introduction of additional negative charges through phosphorylation of the components of the destruction complex increases the binding affinity to the positively charged groove of β-catenin [111]. It is assumed that CK1 and GSK1 are primarily responsible for the phosphorylation of Axin, thereby influencing the binding capacity between Axin and β-catenin. Higher binding efficacy allows for better N-terminal β-catenin phosphorylation by the same kinases, namely CK1 and GSK3 [112,113,114].

The counterpart to Axin phosphorylation by CK1 kinase is Protein Phosphatase 1 (PP1). Elevated activity of PP1 results in Axin being predominantly dephosphorylated, thereby impairing the assembly of the destruction complex, and particularly the direct binding to β-catenin. This leads to enhanced stabilization of β-catenin and subsequent activation of the Wnt signaling pathway. Targeted inhibition of PP1 within this pathway may present therapeutic opportunities for conditions characterized by heightened β-catenin signaling [115].

#### 3.2.6. Phosphorylation of TCF/LEF by Nlk and Casein Kinases

Nemo-like kinases (Nlks) have already been established as crucial regulators of the canonical Wnt signaling pathway [116,117,118]. Nlks function as negative modulators of the transcriptionally active TCF/LEF/β-catenin complex. Notably, Nlks phosphorylate TCF4 at the residues T178 and T189 while LEF-1 is phosphorylated at the residues T155 and S166. This consequently attenuates the binding capacity of the entire Tcf/Lef/β-catenin complex to the negatively charged DNA [116]. In contrast, alternative studies have suggested that the phosphorylation of Lef-1 in neural progenitor cells may positively modulate the Wnt signaling pathway [119]. Therefore, while the inhibitory role of Nlks has conventionally been regarded as a dogma, the regulatory mechanism seems to exhibit greater complexity, manifesting distinct effects in various cell types and developmental stages. However, investigations on suppressing Nlk expression remarkably promoted the proliferation of non-small-cell lung cancer (NSCLC) cells, indicating Nlks to be crucial regulators of the transcriptional activity of the Tcf/Lef/β-catenin complex [120].

Moreover, it is observed that Tcf/Lef also serves as a substrate for several other ubiquitously expressed kinases. Exemplarily, phosphorylation of Tcf3 by CK1 augments the binding to β-catenin, whereas GSK inhibits the interaction of Tcf3 with β-catenin [121].

### 3.3. Dysregulation of Non-Canonical Wnt Signaling

Numerous studies have been dedicated to explore the dysregulation of the canonical Wnt signaling pathway and its underlying mechanisms across diverse tumor types [122,123,124]. Conversely, the non-canonical pathway remains less extensively investigated in this context. The involvement of the non-canonical Wnt pathway in cancer development is intricate and complex. This is also attributed to the fact that dysregulation of the non-canonical pathway results in a broader spectrum of pathological conditions. Malformations manifest during embryonic developmental stages, encompassing diseases such as Robinow syndrome, autism, epilepsy, neural tube defects, and numerous others.

In cancer, it is generally acknowledged that dysregulation can lead to elevated rates of epithelial–mesenchymal transition (EMT). Enhanced migration and motility of cancer cells are primarily contributing to cancer metastasis. In the subsequent section, we will examine the current scientific data and elucidate the causative factors for the dysregulation of the non-canonical Wnt signaling pathway.

#### 3.3.1. Wnt/PCP Pathway

The non-canonical Wnt pathway, as described above, involves the sequential activation of various factors on a post-translational basis (e.g., phosphorylation). Mutations or downregulations of these factors within the signaling cascade generally exert a tumor-suppressive effect on cancer cells [125]. The promotion of tumor progression and aggressiveness is mainly attributed to the excessive or uncontrolled activation of the non-canonical Wnt signaling pathway.

Activation of Wnt/PCP signaling is initiated by the binding of non-canonical Wnt ligands (e.g., Wnt5a or Wnt11) to the FZD receptor, which leads to the recruitment and activation of Dvl. As a scaffold and activator protein, Dvl facilitates the further activation of downstream effector proteins, including Rho family GTPases and c-Jun N-terminal kinase (JNK) [126]. These effector proteins modulate the actin cytoskeleton organization to promote cellular motility [127]. Given the pivotal role of Wnt/PCP signaling in coordinating cell migration, aberrant Wnt/PCP pathway activity may significantly contribute to certain tumor malignancies. Upregulation of core Wnt/PCP components has been documented to enhance cell migration, invasion, and metastasis in various tumor types, including breast cancer [125,128,129], prostate cancer [130], gastric cancer [131,132], colorectal cancer [133,134], and glioblastoma [127].

**Figure 5:**
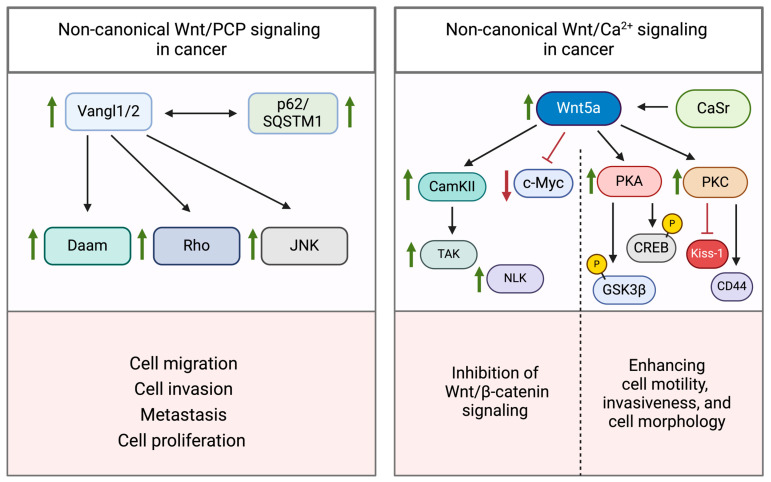
Non-canonical Wnt/PCP and Wnt Ca^2+^ signaling and their implications in cancer. In Wnt/PCP, overexpression of the Vangl1/2 receptor can have activating effects on downstream GTPases and kinases, which ultimately lead to typical cancer characteristics, including cell migration, cell invasion, metastasis, and cell proliferation. The Wnt/Ca^2+^ signaling pathway frequently shows elevated Wnt5a levels in diverse cancer cells. Depending on the tissue type, elevated Wnt5a levels can either act as tumor suppressive (by inhibiting canonical Wnt/β-catenin signaling) or as proto-oncogenic (by inducing cell motility, invasiveness, and cancer cell morphology).Black arrows indicate a direct influence of a protein on its downstream target. Green arrows indicate an increase in activity, while red arrows show a decrease in activity. This illustration was created with BioRender.com (accessed on 24 July 2024).

The regulation of the Wnt/PCP pathway is intricately linked to the activity of the involved kinases. An abnormally elevated activity can thereby promote carcinogenic developments. Next to the FZD receptor, there are other transmembrane proteins equally contributing to the regulation of the Wnt/PCP pathway. The transmembrane proteins Vangl1 and Vangl2 (homologs of Drosophila Van Gogh) play a crucial role in the regulation of polarized cellular behavior [135]. In several cancer types, including breast cancer, ovarian cancer, and uterine corpus endometrial carcinoma, Vangl2 was found to be significantly upregulated (Figure 5; left) [136]. Vangl upregulation is also correlated with aggressive tumor behavior. Ultimately, the Vangl transmembrane proteins, similar to the FZD receptor, exert their activating effects through the Rho GTPase family, c-Jun kinase, Daam1, and other kinases that regulate the polarization of the cytoskeleton [137]. Furthermore, the p62/SQSTM1 protein has been identified as a scaffold protein and Vangl2 interaction partner. This protein complex is capable of activating JNK signaling and thereby promoting proliferation in cancer cells [138].

Not being a direct regulator but acting in conjunction with the Wnt/PCP pathway is PRICKLE1, a key modulator in regulating the motility of cancer cells. This protein forms a complex with the pro-migratory serine/threonine kinase MINK1 [139]. Depletion of either PRICKLE1 or MINK1 in breast cancer cells has been demonstrated to reduce cell motility by promoting the formation of dense actin bundles and cellular spreading. Furthermore, the interaction between PRICKLE1 and RICTOR, a component of the mammalian target of rapamycin complex 2 (mTORC2), is integral to the subsequent activation of the serine/threonine kinase AKT [140]. The mTOR-AKT pathway plays a critical role in various cellular processes, including cell migration and consequently determining the course of tumor progression and metastasis [141]. In conclusion, the PRICKLE1-MINK1-mTORC2 complex intricately regulates AKT phosphorylation, contributing to the migratory potential of cancer cells [139].

Especially the disorder of the Wnt/PCP pathway and related regulatory proteins emphasize its dual role in cancer pathophysiology. While upregulation of Wnt/PCP components enhances cell migration, invasion, and metastasis in various tumor types, including breast cancer [125,128,129], prostate cancer [130], gastric cancer [131,132], colorectal cancer [133,134], and glioblastoma [127], depletion of PRICKLE1 and MINK1 has been linked with reduced cell motility in breast cancer [141]. Aberrant Wnt/PCP signaling can, thus, have both growth-promoting and growth-suppressing effects depending on the cancer type and regulatory proteins within this pathway.

#### 3.3.2. Wnt/Ca^2+^ Pathway

As already stated, the Wnt/Ca^2+^ signaling pathway is mainly involved in ventral cell fate and tissue separation during embryogenesis. This pathway is also activated through Wnt ligands (e.g., Wnt5a) which trigger the transient increase in intracellular Ca^2+^ and, with that, the unraveling of a series of activated kinases within this signaling cascade (Figure 5; right). Nevertheless, conflicting findings exist in the literature regarding the tumor-suppressive or proto-oncogenic role of the Wnt5a ligand. This dichotomy has, however, been observed across disparate tissues.

With regard to calcium signaling, Wnt5a exhibits tumor suppressor function in several tissue and tumor types, such as neuroblastoma [142,143], esophageal squamous cell carcinoma [144], acute myeloid lymphoma [145,146,147], breast cancer [148,149,150], thyroid carcinoma [151], and colon carcinoma [152,153]. Conversely, it assumes a proto-oncogenic role in prostate cancer [154,155], melanoma [156,157], breast cancer [158], and pancreatic cancer [159,160,161].

**Table 2:** Genes and their respective proteins involved in tissue-dependent malignancies.

Gene	Tumor Pro-Oncogene /Suppressor	Malignancy (Tissue)	References
CTNNB1 (β-catenin)	Proto-oncogene	Hepatocellular carcinoma, pancreatic cancer, colorectal cancer, gastroesophageal/junction carcinoma, gastric adeno- carcinoma	[57,58]
APC	Suppressor	CRC, uterine endometrial cancer, stomach cancer, skin cutaneous melanoma	[61,62]
AXIN1	Suppressor	HCC uterine endometrial cancer	[61,65]
AXIN2	Suppressor	Colorectal cancer	[61,65]
RNF43	Suppressor	Colorectal cancer, endometrial cancer	[67]
ZNRF3	Suppressor	Uterine and skin cancer	[61]
Nlk	Suppressor	NSCLC (non-small-cell lung cancer)	[120]
Frizzled receptor	Proto-oncogene	Breast cancer	[125,128,129]
		Prostate cancer	[130]
		Gastric cancer	[131,132]
		Colorectal cancer	[133,134]
		Glioblastoma	[127]
Vangl2	Proto-oncogene	Breast cancer, ovarian cancer, uterine corpus endometrial carcinoma	[137]
PRINCKLE1	Suppressor	Breast cancer	[141]
MINK1	Suppressor	Breast cancer	[141]
Wnt5a	Suppressor	Neuroblastoma	[142,143]
		Esophageal squamous cell carcinoma	[144]
		Acute myeloid lymphoma	[145,146,147]
		Breast cancer	[148,149,150]
		Thyroid carcinoma	[152]
		Colon carcinoma	[152,153]
Wnt5a	Proto-oncogene	Prostate cancer	[154,155]
		Melanoma	[156,157]
		Breast cancer	[158]
		Pancreatic cancer	[159,160,161]

Wnt5a exerts its tumor suppressor effect in many ways but typically has the same outcome, which is the repression of the Wnt/β-catenin pathway. In thyroid cancer, Wnt5a downregulated c-myc, which in turn is a well-established proto-oncogene activating the Wnt/β-catenin pathway [151]. Furthermore, the transfection and overexpression of Wnt5a resulted in diminished motility and invasiveness, which was concomitant with the activation of the Ca^2+^/CaMKII pathway. CaMKII, a major component of the Wnt/Ca^2+^ signaling pathway, phosphorylates β-catenin independently of GSK3, facilitating its degradation. In this context, it was shown that activated PKC (influenced by the cytosolic Ca^2+^ influx), is also capable of antagonizing β-catenin-dependent canonical Wnt signaling by engaging the CaMKII-TAK1-Nemo-like kinase (NLK) pathway, leading to the phosphorylation of TCF [52]. Additionally, it can interfere with the nuclear factor of activated T cells (NFAT)-mediated transcriptional regulation. Both ways result in a GSK3-independent degradation of β-catenin [162].

Studies on colorectal cancer cell lines revealed that constitutively active β-catenin in colon cancer cell lines, arising from inactivating mutations within the β-catenin destruction complex, undergoes degradation in the presence of extracellular Ca^2+^ [153]. In particular, extracellular Ca^2+^ was found to activate calcium-sensitive receptors (CaSRs) in intestinal epithelial cells, leading to the transcription and translation of Wnt5a. Upon secretion, Wnt5a engages in autocrine signaling, activating the Wnt5a/Ror2 pathway and subsequently inducing the degradation of β-catenin [152,153]. In contrast, Wnt5a overexpression has been observed to exert anti-apoptotic effects [163]. Wnt5a induces PKA-mediated phosphorylation of GSK3β and CREB. Phosphorylation-induced inactivation of GSK3β facilitates the nuclear translocation of β-catenin, which, in collaboration with CREB, orchestrates the transcription of genes promoting cell survival.

In independent investigations, Wnt5a has demonstrated the ability to enhance metastasis in various cancer cell lines. For instance, it has been documented that Wnt5a induces invasiveness in breast cancer cell lines by acting in tandem with the Wnt/β-catenin pathway [128]. Wnt5a signaling was upregulated in melanoma cells, directly enhancing cell motility, invasiveness, and cell morphology through actin cytoskeleton reorganization. These effects were predominantly mediated by PKC, which is directly influenced by Ca^2+^ increment [128,164,165]. PKC initiates downregulation of metastasis suppressors Kiss-1, upregulation of metastasis-associated CD44, and initiation of epithelial-to-mesenchymal transition [166]. In prostate cancer cell lines, Wnt5a expression was upregulated due to epigenetic influences that rendered the cancer cells highly invasive [167]. It is apparent that the non-canonical Wnt signaling pathway is intricately interconnected with essential cellular signaling pathways, underscoring the challenge of identifying distinct molecular causes for the development of Wnt signaling-related tumors.

## 4. Targeting the Wnt Signaling Pathway in Cancer Therapy

### 4.1. Targeting the Wnt Signaling Pathway on Distinct Cellular Levels

The Wnt signaling pathway is involved in various levels of cellular processes, making it a prime target for cancer therapeutics. The pathway’s regulation occurs at three distinct cellular levels, which makes the Wnt signaling pathway so important for multiple targets to interfere with:

(1) Extracellular and Membrane Levels: The activation of the Wnt pathway is influenced by the presence of Wnt ligands and the expression of receptors such as Frizzled (Fzd) and co-receptors LRP5/6. Preventing the interaction of Wnt ligands with their receptors effectively blocks the signal transduction and subsequently reduces tumor growth and metastasis. Niclosamide, for instance, is a pharmaceutical compound belonging to the class of anthelmintics. It is a salicylamide derivative that is normally used for the treatment of parasitic worm infestations and mollusks. Interestingly, it was found that niclosamide inhibits the Wnt/β-catenin signaling pathway through several mechanisms: It enhances the internalization of the Wnt receptor Frizzled 1 (Fzd1) [168], promotes the degradation of the Wnt co-receptor LRP6 [169], suppresses the expression of the Wnt signaling regulator Dishevelled 2 (Dvl2) [170] and inhibits the formation of the β-catenin/TCF complex [171].
(2) Cytoplasmic Level: At the cytoplasmic level, the stability and concentration of beta-catenin are controlled by components like APC, Axin, and the phosphorylation status of COX-2. Therapeutics that stabilize Axin or other components of the destruction complex, promote the degradation of β-catenin. Inhibition of β-catenin effectively reduces Wnt signaling activity and thereby cell migration and proliferation. Such compounds include the small molecule pyrazole-4-carboxamide (YW2065) [172], which is listed and discussed below.
(3) Nuclear Level: In the nucleus, Wnt signaling regulates gene transcription through factors such as LEF/TCFs, CBP, c-Myc, and cyclin D1, which are essential for cell proliferation and differentiation. Blocking the interaction between β-catenin and CBP leads to reduced β-catenin-mediated transcription. This approach has shown promise in preclinical studies, particularly in enhancing the efficacy of cytotoxic and targeted therapies. Such compounds include the small molecule Foscevivint (PRI-724), which is listed and discussed below [173].

### 4.2. Therapeutic Classes

In recent years, research has increasingly focused on inhibiting the Wnt/β-catenin pathway due to its extensive range of potential targets, aiming to limit cell proliferation in tumor cells. This long-standing research has led to the development of various strategies, some of which have been mentioned above. Consequently, this has resulted in the emergence of different classes of therapeutic agents, which will be discussed in further detail below.

#### 4.2.1. Natural Compounds

Curcumin, a natural compound from turmeric, demonstrates a broad spectrum of pharmacological activities, including antioxidant, anti-inflammatory, hepatoprotective, antidepressant, anti-arthritic, antidiabetic, and antitumor properties [174]. Curcumin especially exhibits an anti-proliferative effect on various cancer cell lines by inhibiting Wnt signaling activity and its downstream mediator cyclin D1 [175]. So far, the exact mechanism of how curcumin inhibits the Wnt/β-catenin signaling is not known. However, clinical trials already exist, that evaluate the efficacy of curcumin in combination with distinct standard neoadjuvant radiotherapy drugs in treating stage I colorectal cancer (CRC) [176]. Genistein, another natural compound derived from soybeans, inactivates Wnt signaling by up-regulating GSK3β and E-cadherin, thereby reversing the resistance to fluoropyrimidine and platinum compounds [177].

#### 4.2.2. Small Molecules

Research on small molecule inhibitors targeting the Wnt/β-catenin pathway has identified several promising compounds. YW2065 is a small molecule stabilizing Axin-1, in turn stabilizing the β-catenin destruction complex, thereby reducing β-catenin levels and eventually inhibiting Wnt signaling [172]. Another significant inhibitor is Foscevivint (PRI-724), which disrupts the interaction between β-catenin and CBP (cAMP-responsive element-binding protein). By blocking this interaction, PRI-724 promotes the differentiation of cancer stem cells and increases their sensitivity to cytotoxic drugs, enhancing therapeutic efficacy in various cancers [173]. Their preclinical effectiveness has been demonstrated in various types of cancer cells, including head and neck squamous cell carcinoma (HNSCC) [178], hepatocellular carcinoma [179], and neuroendocrine tumor cells [180].

#### 4.2.3. Therapeutic Peptides and Peptide Mimetics

Dickkopf-1 (Dkk-1) is a natural protein that counts as a Wnt pathway inhibitor used in preclinical models to reduce tumor growth and metastasis in cancers like osteosarcoma and multiple myeloma [181,182]. Dkk-1 inhibits β-catenin-dependent Wnt signaling by binding to the LRP5/6 co-receptor and preventing the Wnt ligand–receptor binding. This process enhances the destruction complex stabilization and the subsequent β-catenin degradation. However, it is also reported, that high levels of Dkk-1 can also be associated with a poor prognosis for patients with prostate cancer [183]

CWP232291 is an innovative small peptide mimetic that targets the Wnt/β-catenin pathway by specifically inhibiting the transcription of β-catenin-responsive genes. It effectively reduces the levels of critical genes like survivin and cyclin D1, which play vital roles in cell survival and proliferation. Research has shown that CWP232291 can inhibit the growth of castration-resistant prostate cancer by inducing apoptosis-related endoplasmic reticulum (ER) stress, leading to the degradation of β-catenin. In preclinical models, this compound has been effective in reducing the nuclear accumulation of β-catenin and downregulating its target genes, which restricts cancer cell proliferation and enhances apoptosis [173].

Foxy-5 is a synthetic peptide designed to mimic the activity of WNT5A. Preclinical studies have demonstrated that low levels of WNT5A are associated with a more advanced or metastatic progression in breast and prostate cancers. Consequently, Foxy-5 has shown potential in preventing metastasis to a certain extent by compensating for the deficient WNT5A activity, thereby inhibiting the proliferation of cancer cells [173,184,185].

#### 4.2.4. Monoclonal Antibodies

Monoclonal antibodies, including Vantictumab (OMP-18R5) and Ipafricept (OMP-54F28), which are fully humanized immunoglobulin G2 (IgG2) monoclonal antibodies that bind to Frizzled (FZD) receptors 1, 2, 5, 7, and 8, block canonical Wnt signaling. OTSA101, another humanized monoclonal antibody against Frizzled receptor 10 (FZD10), is labeled with yttrium-90 for targeted radiation delivery, selectively killing cancer cells expressing unnaturally high amounts of FZD10.

#### 4.2.5. Novel Emerging Strategies

Vacuolar-type ATPases (v-ATPases) are proton pumps essential for the acidification of various intracellular compartments, a process critical for numerous cellular functions, including protein degradation, membrane trafficking, and autophagy [186,187]. Recent research has underscored a notable link between v-ATPases and the Wnt signaling pathways [188,189,190]. v-ATPases regulate Wnt signaling by modulating the acidification of intracellular compartments, which are important for the correct trafficking and processing of Wnt receptors and ligands. V-ATPase subunits are highly expressed in distinct tumor entities, including colorectal, breast, prostate, liver, ovarian, and pancreatic cancer cells [191,192,193,194]. V-ATPases play a significant role in the endocytosis and recycling of LRP6. Inhibition of v-ATPase activity can lead to the accumulation of Wnt receptors within endosomes, hindering their proper recycling, and thereby impairing Wnt signaling [195]. This inhibition strategy has emerged as a potential therapeutic approach for cancers characterized by aberrant Wnt signaling. v-ATPase inhibitors, such as bafilomycin and concanamycin, have been shown to disrupt Wnt signaling, resulting in decreased cancer cell proliferation and increased apoptosis [196]. Additionally, v-ATPases are crucial for the lysosomal degradation of the β-catenin destruction complex. Therefore, v-ATPase inhibitors enhance the stabilization of the destruction complex and the degradation of β-catenin, leading to the suppression of Wnt signaling.

Tankyrases, including Tankyrase 1 and Tankyrase 2, are poly(ADP-ribose) polymerases (PARPs) that are integral to various cellular functions, such as telomere maintenance, mitosis, and the modulation of signaling pathways, including the Wnt/β-catenin pathway [197]. These enzymes catalyze the addition of ADP-ribose polymers to the protein Axin, which targets it for ubiquitination and subsequent proteasomal degradation. Inhibition of tankyrase activity by compounds such as XAV939 reduces the poly(ADP-ribosyl)ation of Axins, thereby increasing its stabilization and also enhancing the stabilization of β-catenin. In summary, inhibiting tankyrases results in the suppression of Wnt signaling [198].

## 5. Conclusions

Since the discovery of the Wnt signaling pathways and the initiation of extensive investigation, numerous studies have substantially advanced our understanding of the intricate mechanisms and their diverse cellular functions. Aberrant canonical Wnt signaling can originate from various causes, including mutations in essential tumor suppressors and imbalances in kinase protein levels and activities, leading to severe consequences. It not only influences embryonic development but also plays a role in ontogenesis and aggressive characteristics of neoplastic cells. The canonical Wnt/β-catenin pathway stimulates the expression of genes responsible for cell proliferation, survival, differentiation, and cell migration. The non-canonical Wnt signaling pathway orchestrates convergent extension and tissue mobility. It greatly contributes to the motility of cancer cells during metastasis. Nonetheless, different tissue and tumor types exhibit considerable variability in the Wnt pathway mechanisms, underscoring the complexity of the entire network of interdependent regulating proteins. Nonetheless, the range of the Wnt signaling complexity allowed for the development of a variety of anti-tumoral therapeutics for multiple targeting. Wnt signaling is thus one of the most aberrant and significant signaling pathways in cancer.

## Figures and Tables

**Table 1:** Distinct Wnt signaling pathways and their respective receptor–Wnt ligand bindings.

Wnt Signaling Pathway	Receptors	Wnt-Ligands
Canonical Wnt/β-catenin Signaling pathway	LRP5/6 and FZD	Wnt1
		Wnt2
		Wnt2b
		Wnt3
		Wnt3a
		Wnt4
		Wnt5a
		Wnt6
		Wnt7a
		Wnt9a
		Wnt10a
		Wnt10b
Non-canonical Wnt/PCP Signaling pathway	ROR1/2, RYP, PTK7 and FZD	Wnt1
		Wnt2
		Wnt5a
		Wnt5b
		Wnt11
Non-canonical Wnt/Ca^2+^ Signaling pathway	ROR1/2, RYP, PTK7 and FZD	Wnt1
		Wnt2
		Wnt5a
		Wnt5b
		Wnt11

## Data Availability

No applicable.
